# The Release of Pollutants through the Bleeding of Cemented Phosphogypsum Backfill: Link to Protocols for Slurry Preparation

**DOI:** 10.3390/ma15207126

**Published:** 2022-10-13

**Authors:** Chendi Min, Ying Shi, Yanan Zhou, Zhixiang Liu

**Affiliations:** School of Resources and Safety Engineering, Central South University, Changsha 410083, China

**Keywords:** bleeding water, phosphogypsum, cemented backfill, pollutant, backfill slurry

## Abstract

The present study investigated the effects of protocols for slurry preparation on the release of pollutants into bleeding water from cemented phosphogypsum (PG) backfill. Backfill slurry was prepared using four different protocols in which different parameters varied, including binder/PG ratio, solid concentration, binder type and mixing procedure. The concentrations of phosphate, fluoride and sulfate and the pH values of the obtained bleeding water were measured. The results demonstrated that the slurry preparation protocols affected the quantities of pollutants through the concentrations of pollutants in bleeding water and the bleeding rate. On the one hand, the binder/PG ratio was the key factor influencing the concentrations of all pollutants in bleeding water. Comparatively speaking, the binder type and mixing procedure had an obvious influence on the fluoride concentration but had little influence on the phosphate and sulfate concentrations in the bleeding water. On the other hand, the protocols for slurry preparation affected the bleeding rate by determining the water retention and water content of the backfill slurry. The most effective protocol for slurry preparation for cemented PG backfill could reduce the bleeding rate and enhance the immobilization of pollutants, minimizing the phosphate concentration in bleeding water to below 0.2 mg/L. However, it appeared that the fluoride concentration was still tens of milligrams per liter (over the limit of 10 ten milligrams per liter), to which attention should be paid.

## 1. Introduction

Cemented backfill is the most efficient approach for recycling the solid waste produced in mining and mineral processes and for reducing rock instabilities caused by high excavation-induced stress [1,2,3,4]. In cemented backfill, backfill slurry is prepared by mixing the aggregate (solid wastes), binder and water in certain proportions. After being mixed homogeneously, the slurry is transported to the stopes through the pipeline. To promote fluidity, excessive water is usually added when preparing backfill slurry [5]. As a result, the water in the backfill slurry can be characterized as free water and water on the particle surface layer [6]. When the slurry is placed in the stopes, the free water gradually separates from the backfill slurry, accumulating as bleeding water [7]. Previous studies showed that the bleeding property is widespread in backfill slurry [8,9]. However, due to the use of solid waste as raw material, the bleeding water derived from the backfill slurry might contain large quantities of pollutants, thus threatening the surrounding environment. Clearly, the bleeding of backfill is an important pathway for the release of pollutants, which is often overlooked.

As an efficient method for phosphogypsum (PG) management, cemented PG backfill is applied in several mines in China, including the Kaiyang phosphate mine, the Jinchuan nickel mine and the Huangmailing phosphate mine [2,10,11,12]. PG is a type of solid waste produced via phosphoric acid production, which can be simplified with the following equation [13,14]:

Ca_5_(PO_4_)_3_F + 5H_2_SO_4_ + 10H_2_O → 3H_3_PO_4_ + 5CaSO_4_(H_2_O)_2_ + HF + heat (1)

According to Equation (1), calcium sulfate dihydrate (CaSO_4_·2H_2_O) accounts for more than 90% of PG and the main impurities in PG include H_3_PO_4_, HF and unreacted H_2_SO_4_ [15]. Due to the strong demand for phosphoric acid in the fertilizer industry, the global production of PG is huge, especially in the main phosphate rock-producing countries, including China, the USA and Morocco [16]. For instance, the annual PG production in China, the USA and Morocco is approximately 75 million tons, 40 million tons and 15 million tons, respectively [17,18,19]. Due to the lack of a large-scale utilization method, only 15% of the produced PG is reused to produce agricultural fertilizers [20], set retarders for cement [21,22], soil amendments [23], plaster [24,25] and cementitious materials [26]. The remaining 85% of PG is directly stockpiled, resulting in serious environmental damage to soil, water and the atmosphere [27]. On the other hand, increasingly stringent environmental regulations in China result in the high maintenance costs of stockpiled land, reducing the profitability of the phosphate fertilizer industry. It was reported that cemented PG backfill could utilize 60% of the produced PG [2], which could greatly reduce the costs of PG management. As a waste-based material, the pollutant solidification/stabilization (S/S) efficiency of cemented PG backfill raises concerns. An SPLP test on cemented PG backfill showed that the heavy metals in leachate were within the limit values of Chinese standard DZ/T 0290-2015 [2]. A three-step sequential extraction test indicated that cemented PG backfill effectively solidified/stabilized the metals in PG [28]. The phosphate in the leachate of cemented PG backfill was also efficiently immobilized [29]. However, previous studies mainly focused on leachate of hardened cemented PG backfill rather than on bleeding water.

**Figure 1:**
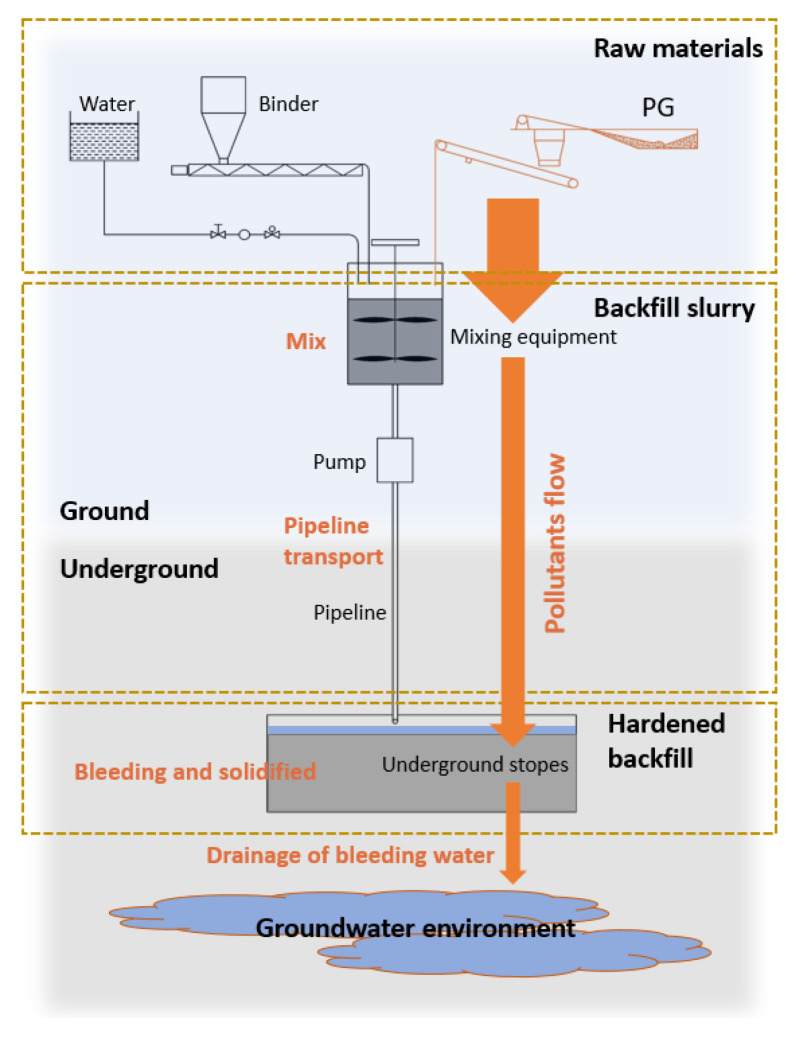
How pollutants are released into the surrounding environment via bleeding water.

The release of pollutants through bleeding water could be very different from that of hardened backfill. The bleeding phenomenon occurs immediately after the backfill slurry is placed into the stopes before the S/S process is fully effective. Thus, compared with leachate of hardened backfill, bleeding water could pose a high risk for its release of pollutants. The contents of phosphate, fluoride and sulfate in PG are rather high; active PO_4_^3−^, F^−^ and SO_4_^2−^ could be the main pollutants in bleeding water. Moreover, due to the specific physiochemical properties of the aggregate PG, the water content in cemented PG backfill is rather high to obtain the desired slurry fluidity. Backfill slurry is usually prepared with a solid concentration of 40–65% [2,30,31]. Previous studies showed that the slurry of cemented PG backfill has a relatively high bleeding rate [29,31,32]. For instance, Zhou et al. showed that the bleeding rate was 25–51%, depending on the content of soluble phosphate in PG [29]. The high bleeding rate could further increase the damage of bleeding water to the surrounding environment. Figure 1 illustrates how the pollutants are released into the surrounding environment via bleeding water. The backfill slurry is prepared and firstly transported to the underground stopes. Then, the backfill slurry bleeds and solidifies, providing strength for mining. For cemented backfill using active waste as aggregate such as PG, part of the pollutants in PG are immobilized in the inner backfill matrix via the hydration of binder, and the rest migrate from the backfill slurry to the bleeding water. The massive bleeding water can be drained and enter the groundwater environment. As a result, the pollution caused by cemented PG backfill via bleeding water is worthy of attention.

It is widely known that the protocols for slurry preparation (binder/aggregate ratio, solid concentration, etc.) have an effect on the strength of hardened backfill. Moreover, previous studies indicated that the bleeding property also varies with the protocols for slurry preparation, which could affect the release of pollutants. Wang et al. found that the bleeding rate of backfill slurry with a low solid concentration of 74% was 20.44% higher than that obtained with a solid concentration of 82% [33]. Furthermore, the binder type could be a factor affecting the bleeding rate. Yao et al. showed that when the fly ash content in the binder was increased from 0 to 20%, the bleeding rate of the backfill slurry increased from 2.45 to 6.07% [34]. In addition, it was also observed that water-reducing agents could lead to a change in the bleeding rate [35]. However, previous studies only used the bleeding rate as an index to evaluate the water retention and stability of the slurry. There are few studies considering the environmental pollution caused by bleeding water derived from slurry prepared using different protocols.

The present study aimed to investigate the effect of different protocols for slurry preparation on the release of pollutants from cemented PG backfill slurry into the bleeding water. Backfill slurry was prepared using different binder/PG ratios, solid concentrations, binder types and mixing procedures. The bleeding rate was tested and the bleeding water was sampled. The release of pollutants was characterized based on the concentrations of PO_4_^3−^, F^−^ and SO_4_^2−^ in bleeding water and the quantities of pollutants released per ton of solid in backfill. This study enhanced the understanding of the environmental damage caused by the bleeding of cemented PG backfill.

## 2. Materials and Methods

### 2.1. Materials

**Figure 2:**
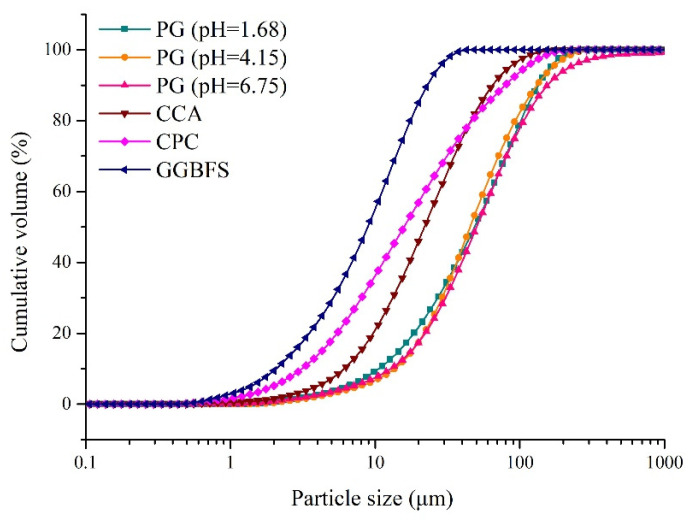
Particle size distribution of PG, CCA, PC and GGBFS [30,37].

**Figure 3:**
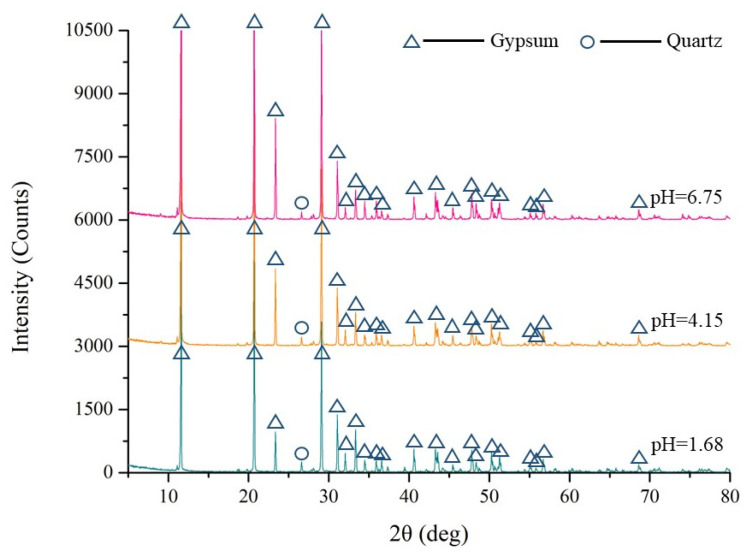
X-ray diffraction analysis of PG.

PG was collected from a backfill plant in Guizhou, China. Considering that the pH value of PG is in the range of 1–7, three batches of PG with pH values of 1.68, 4.15 and 6.75 (measured by mixing PG and deionized water in a 1:1 ratio) were collected in this study. PG was used as an aggregate for cemented backfill. A composite cementitious agent (CCA; prepared with phosphorous slag, fly ash cement clinker and slaked lime [36]), 32.5R composite Portland cement (CPC) and S95 ground granulated blast furnace slag (GGBFS) were used as binders in this study. Figure 2 and Table 1 show the particle size distribution and physical properties of the raw materials, respectively, and indicate that the three batches of PG were coarser than the binders. In particular, GGBFS had the finest particle size. Table 2 and Figure 3 show the main chemical components (measured using X-ray fluorescence; Bruker, Billerica, MA, USA) and crystal phases (measured using advance D8 X-ray automatic diffractometer; Bruker, Billerica, MA, USA) of the PG samples, respectively. The results indicated that the main component of PG was gypsum. In addition, it was observed that the PG sample with a low pH value contained more P_2_O_5_ and F.

### 2.2. Methods

#### 2.2.1. Preparation of Backfill Slurry

Batch A: To investigate bleeding water from backfill slurry with different binder/PG ratios, the binder/PG ratios were set to 1:2, 1:3, 1:4; 1:5, and 1:6. PG, binder and tap water were mixed for 30 min at a constant solid concentration of 50%. The PG with a pH value of 1.68 and CCA were used as aggregate and binder, respectively.

Batch B: To obtain bleeding water from the backfill slurry with different solid concentrations, the backfill slurry was prepared at solid concentrations of 40, 45, 48, 50, 52, 55, 58 and 60% with a mixing time of 30 min. The binder/PG ratio was 1:4. The PG with a pH value of 4.15 and CCA were used as aggregate and binder, respectively.

Batch C: This batch was used to obtain bleeding water from backfill slurry with different binder types. GGBFS was used to partially substitute PC as the binder. The GGBFS replacement proportions were 0, 20, 40, 60 and 90%, and the mixing time was 30 min. The binder/PG ratio was 15:85, and the solid concentration of backfill slurry was 60%. The PG with a pH of 6.75 was used as the aggregate.

Batch D: To obtain bleeding water from backfill slurry using different mixing times, the slurry was sampled after mixing times of 5, 15, 30, 45, 60, 120, and 240 min. The binder/PG ratio was 1:4, and the solid concentration was set to 65%. The PG with a pH value of 4.15 and CCA were used as backfill materials, respectively.

#### 2.2.2. Toxic Leaching Test

The toxic leaching test was conducted to evaluate the quantities of impurities in the PG used in this study according to Chinese standard HJ 557-2010 [38]. The three batches of PG (dry mass) and deionized water were mixed in a ratio of 1:10. The mixture was put on a horizontal shaker to shake at 110 rpm/min for 8 h and then was moved to a table for 16 h. The supernatant was collected and filtered through a 0.45 mm filter for further analysis. The concentrations of PO_4_^3−^ (as mg/L P) and SO_4_^2−^ in bleeding water were measured with a spectrophotometer (Shimadzu, Japan). The concentration of F^−^ was measured using a fluorine ion-selective electrode (Leici, Shanghai, China). The pH and total dissolved solids (TDS) were tested using a pH meter (Ohaus, Parsippany, NJ, USA) and a TDS meter (Ohaus, Parsippany, NJ, USA), respectively.

#### 2.2.3. Bleeding Rate

The bleeding rate test refers to Chinese standard GB/T 50080-2016 [39]. The backfill slurry was poured into a container with lid after mixing for the designed time. The supernatant water was aspirated using an injector at 10 min intervals in the first hour and at 30 min intervals thereafter until no bleeding occurred. The bleeding rate was calculated using Equation (2), where *B* is the bleeding rate, *V*_b_ is the volume of bleeding water, *W* is the mass of water used to prepare the backfill slurry, *G*_1_ is the mass of backfill slurry and *G*_2_ is the mass of backfill slurry in the container.

(2) $$B=\frac{V_{b}}{{(W/G}_{1}{)G}_{2}}\times 100$$

#### 2.2.4. Sampling and Chemical Analyses of Bleeding Water

Backfill slurry was collected after the designed mixing times. Homogeneous backfill slurry was injected into the tubes of a centrifuge. The configuration of the centrifuge was set to a rotating speed of 4000× *g* r/min and a rotating time of 2 min. Supernatant water was collected and passed through a 0.45 μm filter for chemical analyses. The pH and the concentrations of PO_4_^3−^, F^−^ and SO_4_^2−^ in bleeding water were measured according to the methods given in Section 2.2.2. The pH of bleeding water reflected the pH of backfill slurry.

#### 2.2.5. Quantities of Pollutants Released Per Ton of Solid in Backfill

The quantities of pollutants released in bleeding water per ton of solid were used to evaluate the migration of pollutants from backfill slurry into the bleeding water. The quantities of pollutants released per ton of solid were calculated using Equation (3), where *Q* is the quantity of a pollutant released per ton of solid, *C*_p_ is the concentration of a pollutant in bleeding water, *B* is the bleeding rate of backfill slurry, *W* is the mass of water used to prepare backfill slurry and *M*_s_ is the mass of the solid in backfill slurry.

(3) $$Q=\frac{C_{p}\left(B\times W\right)}{M_{s}}$$

## 3. Results

### 3.1. Chemical Properties of PG

**Figure 4:**
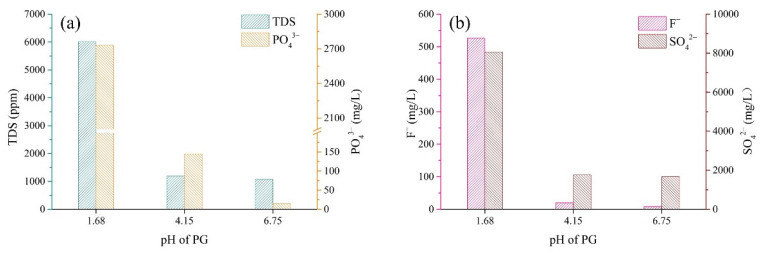
Concentrations of pollutants in leachate of PG with different pH values. (**a**) TDS and PO_4_^3−^; (**b**) F^−^ and SO_4_^2−^.

Figure 4 shows the concentrations of pollutants in the leachate of PG with different pH values. Overall, the pH appeared to be inversely correlated with the TDS value and the concentrations of PO_4_^3−^, F^−^ and SO_4_^2−^ in the leachate of PG, indicating that the PG with a low pH value contained more soluble ions. As the pH of PG increased from 1.68 to 6.75, the concentrations of PO_4_^3−^, F^−^ and SO_4_^2−^ in leachate decreased by 99.5, 98.5 and 79.2%, respectively. Nevertheless, in the case of the PG with a pH of 6.75, the concentration of PO_4_^3−^ was still 29.6 times higher than the limit value (0.5 mg/L) according to the Chinese standard GB 8978-1996 [40]. The results indicated that low pH was closely related to high quantities of soluble pollutants, while high pH values meant fewer pollutants in PG.

### 3.2. Binder/PG Ratio

(1) pH and bleeding rate of backfill slurry

**Figure 5:**
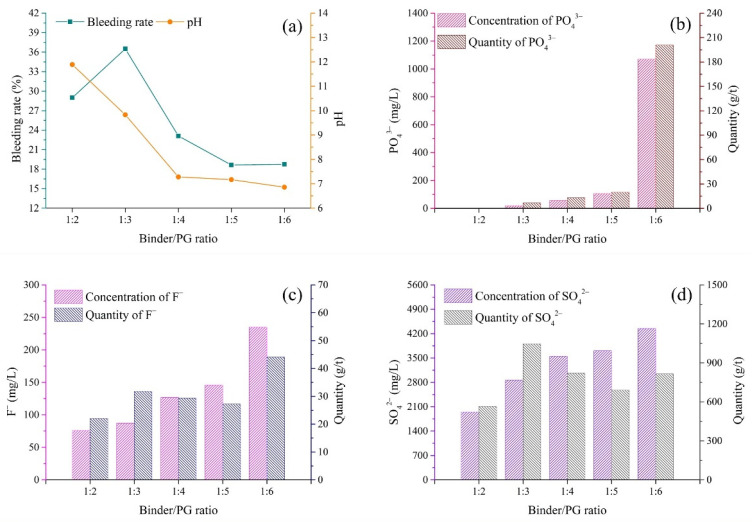
Variations in bleeding rate, pH and pollutants in bleeding water according to different binder/PG ratios of backfill slurry. (**a**) Bleeding rate and pH; (**b**) PO_4_^3−^; (**c**) F^−^; (**d**) SO_4_^2−^.

The binder/PG ratio is a factor determining the strength of hardened backfill. In cemented PG backfill, the binder/PG ratio could also affect the pH and bleeding properties of the backfill slurry. Figure 5a shows the variation in the pH of backfill slurry according to different binder/PG ratios. When the binder/PG ratio varied from 1:2 to 1:4, the pH exhibited a drastic decrease from 11.89 to 7.28. Then, the pH further decreased to 6.86 with the reduction in the binder/PG ratio to 1:6. It can be seen that the presence of more alkali provided by the high content of binder could effectively neutralize the residual acids of PG, while a low content of binder led to low-pH backfill slurry. Because the hydration of the binder usually requires a high pH value, slurry with a binder/PG ratio above 1:3 could smoothly carry out hydration [41].

The variation in the bleeding rate according to the binder/PG ratios is shown in Figure 5a. When the binder/PG ratio was 1:2, the bleeding rate was 29.0%, which was lower than that when the binder/PG ratio was 1:3. This could be explained by the water retention of the binder being better than that of PG, as this ratio (1:2) contained more binder with fine particles, as shown in Figure 2 [42,43]. When the binder/aggregate ratio varied from 1:3 to 1:6, the bleeding rate showed a gradual decrease from 36.5 to 18.76%, which could be associated with the pH of the backfill slurry. Previous studies showed that low pH means low zeta potential and repulsion force between the particles [44,45]. The poor fluidity induced by a low zeta potential may be related to the low bleeding rate [44,46]. These results indicated that the high amount of acids in the aggregate PG increased the water retention capability, thus hindering the bleeding of the backfill slurry.

(2) Pollutants in bleeding water

The variations in the concentrations of pollutants in bleeding water according to different binder/PG ratios are shown in Figure 5b–d. Overall, the concentrations of three typical pollutants (PO_4_^3−^, F^−^ and SO_4_^2−^) significantly increased with the decrease in binder content. In backfill slurry with a binder/PG ratio of 1:2, the concentration of PO_4_^3−^ was only 0.01 mg/L. When the binder/PG ratio decreased to 1:5, the concentration of PO_4_^3−^ increased to 106.4 mg/L, followed by a drastic increase to 1070.0 mg/L in bleeding water when the binder/PG ratio decreased to 1:6. This increasing rule was also applicable to F^−^ and SO_4_^2−^. The concentrations of F^−^ and SO_4_^2−^ increased by 209.2 and 122.9% with the decrease in the binder/PG ratio from 1:2 to 1:6. The results indicated that a high binder content had a positive effect on the immobilization of pollutants in the backfill slurry. It is worth noting that the SO_4_^2−^ in backfill slurry with pH values higher than 11 (binder/PG ratio of 1:2) could still be released from the dissolution of the aggregate PG because its concentration in bleeding water (about 1500 mg/L) was close to the solubility of calcium sulfate.

The quantities of pollutants released per ton of solid in backfill for different binder/PG ratios are shown in Figure 5b–d. The quantity of PO_4_^3−^ in bleeding water increased from 0.003 g/t to 200.7 g/t in the selected binder/PG ratio range, indicating the acceleration of the release of PO_4_^3−^. However, compared with PO_4_^3−^, the released quantities of F^−^ and SO_4_^2−^ showed different patterns with respect to the binder/PG ratio. Figure 5c,d show that when the binder/PG ratio was 1:3, the quantities of F^−^ and SO_4_^2−^ exhibited rather high values, 31.8 g/t and 1046.5 g/t, respectively, which might be attributed to the high bleeding rate causing the high quantities of pollutants released into the bleeding water. High quantities of F^−^ and SO_4_^2−^ were also observed when the binder/PG ratio was 1:6, which was likely related to high concentrations of F^−^ and SO_4_^2−^ in bleeding water.

### 3.3. Solid Concentration

(1) pH and bleeding rate of backfill slurry

**Figure 6:**
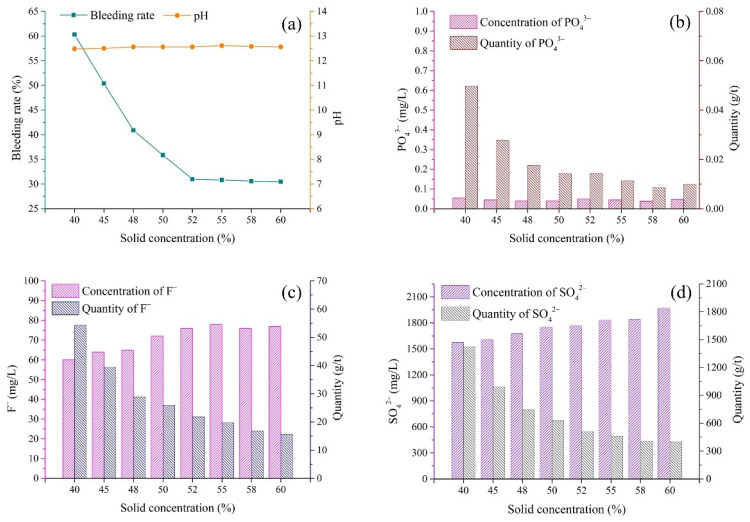
Variations in bleeding rate, pH and pollutants in bleeding water according to different solid concentrations of backfill slurry. (**a**) Bleeding rate and pH; (**b**) PO_4_^3−^; (**c**) F^−^; (**d**) SO_4_^2−^.

As shown in Figure 6a, the pH of the slurry was independent of the solid concentration, while the bleeding rate significantly varied with the solid concentrations. As the solid concentration increased from 40 to 52%, the bleeding rate rapidly decreased from 60.3 to 31.0%. The greater amount of free water introduced via the low solid concentration could be the reason for the high bleeding rate, which was also observed in the study by Yao et al. [42]. When the solid concentration was further increased from 52 to 60%, the bleeding rate only slightly declined from 31.0 to 30.5%. These results indicated that the free water of slurry with a solid concentration of less than 52% more easily accumulated and separated from the slurry and then transformed into the bleeding water.

(2) Pollutants in bleeding water

The concentrations of PO_4_^3−^, F^−^ and SO_4_^2−^ in bleeding water are shown in Figure 6b–d. The concentration of PO_4_^3−^ was measured to be in the range of 0.03–0.06 mg/L, which was much lower than the limit value of 0.5 mg/L [40]. The results indicated that the binder could effectively solidify/stabilize PO_4_^3−^. For F^−^ and SO_4_^2−^, it is clearly shown in Figure 6c,d that the lower the solid concentration was, the lower concentrations of pollutants in bleeding water were. This could be the result of the dilution effect of the greater amount of abundant bleeding water in backfill slurry with a low solid concentration. When the solid concentration increased from 40 to 60%, the concentrations of F^−^ and SO_4_^2−^ gradually increased from 60 to 77 mg/L and from 1575 to 1970 mg/L, respectively.

Figure 6b–d show that the quantities of pollutants released (per ton of solid in backfill) were negatively correlated with the solid concentrations, and their trend was opposite to that of the pollutant concentrations. When the solid concentration increased from 40% to 60%, the quantities of F^−^ and SO_4_^2−^ released per ton of backfill rapidly decreased rapidly from 54.3 to 15.6 g/t and from 1424.6 to 400.2 g/t, respectively. The results indicated that although a greater amount of bleeding water decreased the pollutant concentrations in bleeding water, the high bleeding rate contributed to the release of more pollutants from the backfill slurry into the bleeding water.

### 3.4. Binder Type

(1) pH and bleeding rate of backfill slurry

**Figure 7:**
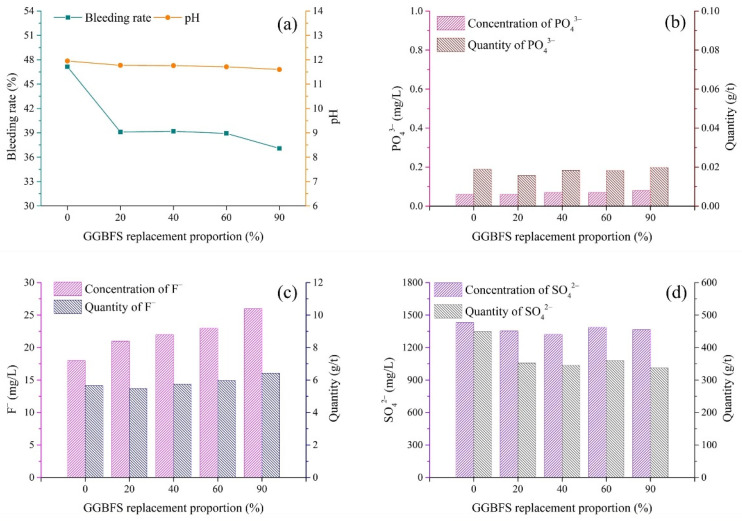
Variations in bleeding rate, pH and pollutants in bleeding water according to GGBFS replacement proportion. (**a**) Bleeding rate and pH; (**b**) PO_4_^3−^; (**c**) F^−^; (**d**) SO_4_^2−^.

Different types of binder showed different immobilization effects on pollutants. The variations in the bleeding rate and pH according to the GGBFS replacement proportion are shown in Figure 7a. When the GGBFS replacement proportion was increased from 0% to 20%, the bleeding rate rapidly dropped from 47.1 to 39.1%. As the GGBFS replacement proportion further increased to 90%, the bleeding water slightly decreased from 38.9 to 37.1%. The results indicated that GGBFS had better water retention capability than PC, as GGBFS was finer than PC (Figure 2) [42,43]. Figure 7a shows that the pH slightly decreased from 11.95 to 11.60 when the GGBFS replacement proportion increased from 0 to 90%, which could be attributed to the neutral characteristic of GGBFS. Nevertheless, the pH values of all slurry samples were always above 11, indicating that the incorporation of alkali was sufficient to activate GGBFS [41].

(2) Pollutants in bleeding water

The concentration of PO_4_^3−^ was in the range of 0.06–0.08 mg/L (Figure 7b), indicating that both PC and PC-GGBFS immobilized PO_4_^3−^ at a low level. Figure 7c shows that the concentration of F^−^ exhibited an increase from 18 mg/L to 26 mg/L in the range of GGBFS replacement proportion from 0 to 90%. The increase in the concentration of F^−^ might be related to the decrease in cement proportion. The results indicated that cement had a better immobilization effect on pollutants than GGBFS. The concentration of SO_4_^2−^, which was mainly derived from the dissolution of CaSO_4_·2H_2_O, exhibited similar values, around 1400 mg/L, for five levels of GGBFS content, as shown in Figure 7d.

As shown in Figure 7b, the quantity of PO_4_^3−^ released was only around 0.02 g/t, which could be the result of the low concentration of PO_4_^3−^. As for F^−^, as shown in Figure 7c, compared with the significant increase in the concentration of F^−^, the release of F^−^ per ton of solid showed only a slight increase for various binder types, which was likely due to the offset effect of the decrease in the bleeding rate. Figure 7d shows that the quantity of SO_4_^2−^ exhibited a decrease in the selected range of GGBFS replacement proportion. This phenomenon could be explained by the combined effect of the stable concentration of SO_4_^2−^ and the decrease in the bleeding rate for various binders.

### 3.5. Mixing Procedure

(1) pH and bleeding rate of backfill slurry

**Figure 8:**
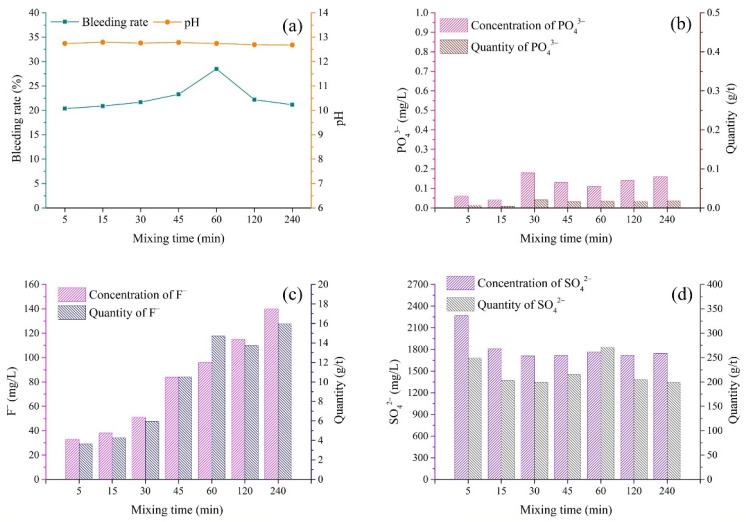
Variations in bleeding rate, pH and pollutants in bleeding water according to mixing time. (**a**) Bleeding rate [37] and pH; (**b**) PO_4_^3−^; (**c**) F^−^; (**d**) SO_4_^2−^.

The slurry of cemented PG backfill requires a rather long mixing time to obtain the proper fluidity for pipeline transport [37]. The properties of the backfill slurry were affected by the mixing time. Figure 8a shows the variations in the pH and bleeding rate of backfill slurry according to different mixing times. The bleeding rate increased over the 5–60 min interval, followed by a decrease when the mixing time was extended to 240 min [37]. The increase in the bleeding rate before 60 min was the result of the instability of the slurry due to mixing, which can reduce the water retention of the slurry [47]. On the other hand, a lower amount of free water induced via hydration with the extension of the mixing time could be the explanation for the decrease in the bleeding rate after 60 min [37]. As for the pH of the backfill slurry, it varied in the small range of 12.68–12.79, indicating that the binder improved the pH of the whole system in the initial stage of mixing.

(2) Pollutants in bleeding water

Figure 8b,c show the changes in the concentrations of PO_4_^3−^ and F^−^ according to the mixing time. It is worth noting that in the first 5 min, the concentrations of PO_4_^3−^ and F^−^ in bleeding water were far below the leachate of PG, indicating that the immobilization of PO_4_^3−^ and F^−^ was accomplished in the first several minutes of mixing. After the first immobilization, the concentrations of both PO_4_^3−^ and F^−^ showed several changes according to the mixing time. For PO_4_^3−^, the concentration increased a little in the first 30 min of mixing and then remained at low levels (about 0.18 mg/L) in the following experimental time intervals. The concentration of F^−^ showed a positive correlation with the extension of the mixing time, as shown in Figure 8c, indicating that the mixing procedure promoted the release of F^−^. The increase in the concentrations of PO_4_^3−^ and F^−^ with the extension of the mixing time could be attributed to the gradual dissolution of Ca_3_(PO_4_)_2_ and CaF_2_, which was related to the consumption of Ca(OH)_2_ via the hydration of the binder. As shown in Figure 8d, the concentration of SO_4_^2−^ showed a relatively high value, 2269 mg/L, in the first 5 min and decreased to 1806 mg/L in 10 min, followed by a relatively stable value after a mixing time of 15 min. The results indicated that the immobilization of SO_4_^2−^ occurred in the first 15 min.

The quantities of pollutants released per ton of solid in backfill are shown in Figure 8b–d. As expected, 1 ton of raw solid in backfill only released 0.005–0.018 g/t of PO_4_^3−^, while the quantity of F^−^ exhibited a gradual increase with the extension of mixing time, as shown in Figure 8c. The quantity of F^−^ was in the range of 4–16 g/t. It is worth noting that the quantities of F^−^ and SO_4_^2−^ after 60 min of mixing time showed relatively high values, which could be explained by the maximum value of the bleeding rate after the mixing time of 60 min.

## 4. Discussion

### 4.1. Release of Pollutants from Backfill Slurry into the Bleeding Water

The quantities of pollutants released were determined by the pollutant concentrations in bleeding water and the bleeding rate.

The pollutant concentrations in the bleeding water were directly determined via the chemical properties of the slurry, such as the pH value. Figure 5 shows that the backfill slurry with low pH released more pollutants into the bleeding water, indicating the presence of high quantities of pollutants in raw materials. More specifically, because of the high content of pollutants in the aggregate PG with low pH (Figure 4), the backfill slurry with a low pH value usually contained more pollutants, which meant that more pollutants were released into the bleeding water, whereas, slurry pH above 11 meant that the pollutants were well immobilized, thus allowing the pollutant concentrations in the bleeding water to remain at relatively low levels. This phenomenon could be explained by the fact that the sufficient Ca^2+^ introduced by the binder transformed active ions such as PO_4_^3−^_,_ F^−^ and SO_4_^2−^ into undissolved forms, reducing the quantities of pollutants in the backfill slurry [29]. In cemented PG backfill, PO_4_^3−^ could be better solidified/stabilized than F^−^ and SO_4_^2−^. To be more specific, PO_4_^3−^ in the bleeding water could be minimized to below 0.2 mg/L when the pH value of backfill slurry was increased over 11 (Figure 5, Figure 6, Figure 7 and Figure 8), which was less than the limit value of 0.5 mg/L [40]. As for F^−^, the concentration was tens of milligrams per liter despite the backfill slurry exhibiting a high pH value, which was higher than the limit value of ten milligrams per liter [40]. It was also observed that the concentration of SO_4_^2−^ in the bleeding water was always higher than 1300 mg/L, even though the pH value of the backfill slurry was higher than 11; this was due to the dissolution of the aggregate PG.

The release of pollutants was also affected by the physical properties of the backfill slurry, such as the bleeding rate. For instance, it was observed (Figure 6) that the backfill slurry with a high bleeding rate, 60.3%, released 54 g of F^−^ into the bleeding water per ton of solid in the backfill, which was 3.5 times the quantity released by the backfill slurry with the bleeding rate of 30.5%. It was clear that a high bleeding rate meant more pollutants were released into the environment. It is worth noting that the high bleeding rate meant that more bleeding water was drained, so the pollutants were diluted. For example, as shown in Figure 6, the backfill slurry with a bleeding rate of 30.5% exhibited 77 mg/L F^−^ in the bleeding water, which was 17 mg/L higher than the concentration with a bleeding rate of 60.3%. Nevertheless, considering the combined effect of the pollutant concentration and the bleeding rate, the total quantity of pollutants released was much higher when severe bleeding occurred.

### 4.2. Effect of Preparation Protocols on Chemical Properties of Bleeding Water

The chemical properties of bleeding water, including pH and concentrations of pollutants, varied with the protocols for slurry preparation.

The pH of bleeding water was mainly affected by the pH of the aggregate PG. When low-pH PG (1.68) was used as the aggregate, the binder/PG ratio showed a significant effect on the pH of the bleeding water. As shown in Figure 5, the pH of the bleeding water was in the range of 6.86–12.70. A high binder/PG ratio introduced more alkali, resulting in the improvement of the pH of the bleeding water. However, the backfill slurry prepared with high-pH PG always had high pH, which was due to the fact that less binder was needed to neutralize the residual acid in PG, therefore sufficient OH^−^ was present in the backfill slurry. For instance, when the high-pH PG (4.15 and 6.18) was used as the aggregate in the studied batches (different solid concentrations, binder types and mixing procedures, as shown in Figure 6, Figure 7 and Figure 8), the bleeding water always showed rather high pH values, above 11.

The preparation protocols demonstrated a relationship with the concentrations of pollutants in the bleeding water. As for the binder/PG ratio, high binder dosage could more efficiently solidify/stabilize pollutants, and fewer pollutants were released. As shown in Figure 5, when the binder/PG ratio increased from 1:6 to 1:2, the S/S efficiency of PO_4_^3−^_,_ F^−^ and SO_4_^2−^ was improved by 100%, 67.7% and 30.7%, respectively. For solid concentration, a greater amount of bleeding water, due to the low concentration, could dilute the pollutants, causing low pollutant concentrations. For binder type and mixing time, it seemed that the concentration of F^−^ positively related to GGBFS content and mixing time, while the concentrations of PO_4_^3−^ and SO_4_^2−^ varied little with these two protocols, as shown in Figure 7 and Figure 8.

### 4.3. Effect of Preparation Protocols on Bleeding Rate of Backfill Slurry

The bleeding rate was affected by the preparation protocols in two aspects: (i) the initial water content and (ii) the water retention. On the one hand, it was clear that the initial water content was the critical factor in terms of solid concentration. As shown in Figure 6, when the initial water content increased from 48 to 60%, the bleeding rate increased by about 50%. On the other hand, the water retention was affected by the protocols for slurry preparation. As for the binder/PG ratio, the water retention of the backfill slurry was intensified by the low pH related to the low binder/PG ratio, as shown in Figure 5. As for the binder type, the GGBFS replacement proportion could increase the water retention, which was related to the large specific surface area of the binder. The fine GGBFS particles increased the requirement for water on the surface layer and decreased the proportion of free water, increasing the water retention and reducing the bleeding rate [42,43].

In the cemented PG backfill, the backfill slurry exhibited a rather high bleeding rate ranging from 18.6 to 60.3%, which was much higher than that of the backfill slurry obtained using tailings as aggregates (lower than 15% to improve the roof-contacted filling ratio) [48]. The high bleeding rate could be attributed to the high initial water content that was required to obtain the desired fluidity. Different from the tailings, the specific gravity of PG is much lower [49,50,51]. With the same solid concentration of backfill slurry, the particle number of aggregate PG can be much larger than that of aggregate tailings. The larger number of particles in the cemented PG backfill slurry results in a larger total specific surface area and requires greater water content to ensure fluidity [42]. In practice, in mines, the solid concentration of cemented PG backfill is generally 40–65%, which is much lower than that of cemented tailings backfill (which typically ranges from 70 to 85%) [2,31,48,52,53].

**Figure 9:**
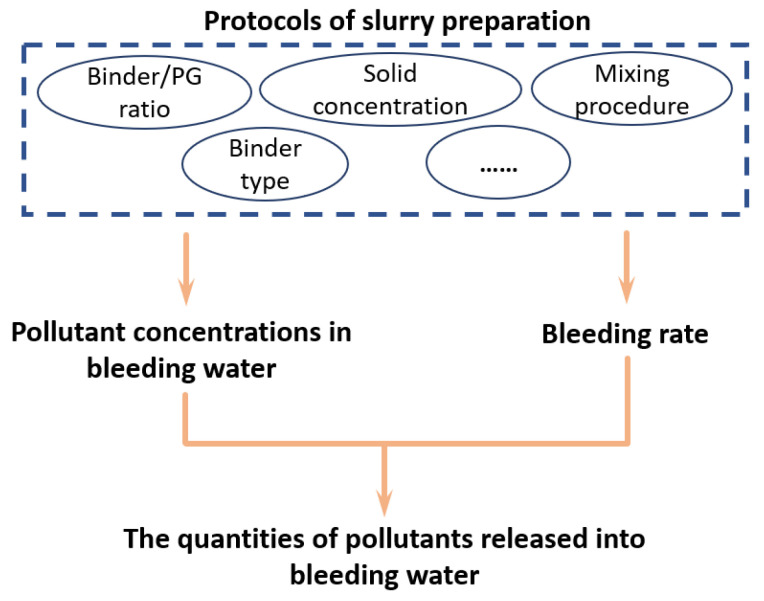
Schematic illustration of how the protocols for slurry preparation affected the release of pollutants from backfill slurry into bleeding water.

A schematic illustration of how the protocols for slurry preparation affected the release of pollutants into the bleeding water is shown in Figure 9. The protocols for slurry preparation affected the concentrations of pollutants in bleeding water and the bleeding rate of backfill slurry. Increasing the pH of the backfill slurry by adding more binder reduced the concentrations of pollutants in bleeding water. On the other hand, increasing the solid concentration and the content of fine raw materials reduced the bleeding water produced. Furthermore, the results of this study indicated that the high pollutant concentrations and the high bleeding rate of the backfill slurry contributed to the release of pollutants into the bleeding water.

## 5. Conclusions

In this study, four different protocols for slurry preparation were considered to investigate the release of pollutants from cemented PG backfill slurry into the bleeding water. Based on the above results, conclusions could be drawn as shown below:

(1) The bleeding water from cemented PG backfill contained PO_4_^3−^, F^−^ and SO_4_^2−^, which pose a risk of damage to the surrounding environment. The high pollutant concentrations and the high bleeding rate resulted in higher quantities of pollutants being released from the backfill slurry;
(2) The pollutant concentrations in the bleeding water could be minimized via proper protocols for slurry preparation, such as increasing the binder/PG ratio. A greater amount of more binder efficiently transformed PO_4_^3−^, F^−^ and SO_4_^2−^ into undissolved forms. The dilution effect induced by the low solid concentration also helped to reduce the pollutant concentrations;
(3) The poor water retention and high initial water content of the backfill slurry increased the bleeding rate. Introducing more fine raw materials into the backfill slurry, such as binder and GGBFS, could bond more surface layer water and increase the water retention. The pH of the backfill slurry is also negatively related to water retention;
(4) Cemented PG backfill could solidify/stabilize PO_4_^3−^ in the bleeding water well when the pH value was higher than 11. However, the concentration of F^−^ in the bleeding water always exceeded the limit value of the national standard (10 mg/L); therefore, further studies are needed for the S/S of F^−^.

## Figures and Tables

**Table 1:** Physical properties of PG, CCA, PC and GGBFS.

Sample	D10 (μm)	D30 (μm)	D60 (μm)	Cu	Cc
PG (pH = 1.68)	10.53	27.40	62.91	6.08	1.13
PG (pH = 4.15)	13.65	29.02	56.38	4.13	1.09
PG (pH = 6.75)	13.56	33.15	62.76	4.63	1.29
CCA	6.12	13.26	27.74	4.53	1.04
CPC	3.33	8.14	22.60	6.79	0.88
GGBFS	2.00	5.55	11.94	5.97	1.29

**Table 2:** Main chemical components of the three batches of PG.

Chemical Component	PG (pH = 1.68) (%)	PG (pH = 4.15) (%)	PG (pH = 6.75) (%)
SO_3_	44.76	42.19	42.05
CaO	34.93	34.04	34.01
SiO_2_	3.23	2.73	2.31
P_2_O_5_	2.61	0.80	0.65
F	0.81	0.43	0.35
Fe_2_O_3_	0.28	0.25	0.27
Ba	0.17	0.17	0.09
MgO	0.14	0.04	0.05
Na_2_O	0.13	0.03	-
K_2_O	0.10	0.08	0.05

## Data Availability

Not applicable.
